# Association between periodontitis and osteoporosis in United States adults from the National Health and Nutrition Examination Survey: a cross-sectional analysis

**DOI:** 10.1186/s12903-023-02990-4

**Published:** 2023-05-02

**Authors:** Jing Peng, Jianming Chen, Yucheng Liu, Jun Lyu, Bin Zhang

**Affiliations:** 1 Department of Orthodontics, Affiliated Stomatology Hospital of Guangzhou Medical University, Guangdong Engineering Research Center of Oral Restoration and Reconstruction, Guangzhou Key Laboratory of Basic and Applied Research of Oral Regenerative Medicine, Guangzhou, 510182 Guangdong China; 2 Department of Clinical Research, The First Affiliated Hospital of Jinan University, Guangzhou, 510630 Guangdong China; 3 Guangdong Provincial Key Laboratory of Traditional Chinese Medicine Informatization, Guangzhou, 510632 Guangdong China

**Keywords:** Periodontitis, Osteoporosis, Menopausal women, NHANES

## Abstract

**Objective:**

This cross-sectional study aimed to investigate the association between periodontitis and osteoporosis among US adults as well as the subgroup of menopausal women.

**Background:**

Periodontitis and osteoporosis are both chronic inflammatory diseases characterized by local or systemic bone resorption. Since they share many risk factors, and the significant decrease in estrogen along with menopause is unfavorable for both diseases, it’s reasonable to assume that there exists some links between the two diseases, especially during the menopause.

**Methods:**

We analyzed data from the National Health and Nutrition Examination Survey (NHANES) 2009–2010 and 2013–2014. Periodontitis (defined according to the CDC/AAP definition) and osteoporosis (assessed by the dual-energy radiation absorptiometry) data were available for 5736 participants, and 519 subjects were enrolled in the subgroup of menopausal women aged 45–60 years old. We used binary logistic regression analysis to examine the association between the two diseases both in crude and fully adjusted model.

**Results:**

In the fully adjusted model, osteoporosis was significantly associated with an increased risk of periodontal disease (OR:1.66, 95% CI: 1.00–2.77) in the whole population. As to the subgroup of menopausal women, osteoporosis group had an adjusted OR of 9.66 (95% CI: 1.13–82.38) for developing severe periodontitis in the fully adjusted model.

**Conclusions:**

Osteoporosis is significantly associated with periodontitis and the association is even more pronounced in menopausal women with severe periodontitis.

**Supplementary Information:**

The online version contains supplementary material available at 10.1186/s12903-023-02990-4.

## Introduction

Periodontitis is a chronic inflammatory disease characterized by progressive destruction of the tooth supporting tissues, typical clinical manifestations including gingival bleeding, formation of periodontal pocket, gingival recession, radiography change of alveolar bone resorption, tooth mobility, or even tooth loss. Globally, severe periodontitis is the sixth most prevalent disease, affecting 11.2% of the population, and the global cost of lost productivity from it has been estimated at $54 billion a year [1, 2]. While periodontitis is unquestionably initiated by bacteria, it is the individual’s host inflammatory response and other superimposed risk factors that ultimately determine the outcome and prognosis of the disease [3].

Osteoporosis is a common skeletal disease characterized by low bone mass and microarchitectural deterioration of the skeleton, with a consequently increasing risk of bone fragility and predisposition to fracture [4]. Since osteoporosis is also a multifactorial chronic disease and defined by the preponderance of systemically and/or locally bone resorption, it shares many risk factors with periodontitis, such as age, lifestyle behaviors (smoking status, alcohol consumption), low body mass index (BMI) and so on [5]. Among other things, menopause occurs on average at 51 years old and it’s characterized by a significant decrease in estrogen, along with suppression in calcium absorption as well as diminished protection from bone resorption [6], which seems to be unfavorable for both periodontitis and osteoporosis. Hence, it’s reasonable to hypothesis that periodontal destruction may be influenced by systemic change of bone mineral density (BMD) induced by osteoporosis, especially during the menopause. However, the exact mechanisms mediating the association between the two diseases remain inconsistent. Many previous researches have revealed a positive relationship between them, but others disagreed [7–9]. The discrepancy might be explained by different size of study population, definition of two diseases and selection of confounders.

Accordingly, we combined dataset from the 2009–2010 and 2013–2014 National Health and Nutrition Examination Survey (NHANES), and aims of our research were to: 1) provide updated estimates of the association between periodontitis and osteoporosis in the fully adjusted model with confounders of sociodemographic variables, lifestyle behaviors, disease-related health conditions and oral examination. 2) assess whether there existed higher Odds ratio (OR) for periodontitis in the subgroup of menopausal women aged 45–60 years old.

## Methods

The NHANES is complex cross-sectional survey of United States non-institutionalized individuals since the 1960s, conducted by the National Center for Health Statistics and Centers for Disease Control and Prevention. It consists of six unique data sets generated in two-year cycles, and a representative sample of about 10,000 participants across the country is enrolled in each cycle [10, 11]. Since our study is a secondary analysis of publicly data which are available at the website (https://www.cdc.gov/nchs/nhanes/index.htm), no additional ethical approval is necessary. Presently, we collected data from cycles of 2009–2010 and 2013–2014 (data for both periodontitis and osteoporosis were only measured at the same time in these two cycles). Considering the stratification and clustering of the design, we used the weighting procedure of wtmec2yr to provide estimates for the entire 4 years, as suggested by the NHANES website. Natural menopause was defined according to the self-reported reproductive health questionnaire. Females aged 45–60 years were regarded as menopausal who answered “no” to the question “Have you had at least one menstrual period in the past 12 months?” and subsequently answered “menopause/hysterectomy” or “menopause/change of life” to the question “What is the reason that you have not had a period in the past 12 months?” [6]. The details of the self-reported reproductive health questionnaire are available on the NHANES website (https://wwwn.cdc.gov/Nchs/Nhanes/).

### Diagnosis of periodontitis

Participants aged 30 years and older with at least one tooth (excluding third molars) underwent a full-mouth periodontal examination by trained and certified dentists at a mobile examination center. Exclusion criteria included self-reported heart transplant, artificial heart valves, congenital heart disease or bacterial endocarditis. Gingival recession and pocket depth (PD) were measured at 6 sites of each tooth, and attachment loss (AL) was calculated as the difference between the two indexes. The classification algorithm of periodontitis follows the Centers for Disease Control and Prevention and American Academy of Periodontology (CDC/AAP) definition: Severe periodontitis was defined as ≥ 2 interproximal sites with ≥ 6 mm AL (not on the same tooth) and ≥ 1 interproximal site(s) with ≥ 5 mm PD. Moderate periodontitis was defined as ≥ 2 interproximal sites with ≥ 4 mm clinical AL (not on the same tooth) or ≥ 2 interproximal sites with PD ≥ 5 mm, also not on the same tooth. Mild periodontitis is defined as ≥ 2 interproximal sites with ≥ 3 mm AL and ≥ 2 interproximal sites with ≥ 4 mm PD (not on the same tooth) or one site with ≥ 5 mm PD [12].

In our research, we used the term "periodontal disease" to generalize the status of mild, moderate and severe periodontitis groups as previous study described [13, 14].

### Diagnosis of osteoporosis

The dual-energy radiation absorptiometry (DXA) was used to examine the BMD of lumbar spine and femoral neck. Osteoporosis and osteopenia were defined in the present study using the WHO criteria [4]. Specifically, osteoporosis was defined as a T-score ≤  − 2.5 at either the femoral neck or the lumbar spine. Among those without osteoporosis, osteopenia was defined as those with T-scores between − 2.5 and − 1.0, and normal was defined as T-score ≥  − 1. T-scores were calculated as (mean BMD _respondent group_—mean BMD _reference group_) / SD _reference group_. The reference group for calculation of the femoral neck consisted of 20–29 white females from the NHANES III report [15]. As there is no internationally recommended reference group for the lumbar spine, the reference group consisted of 24 normal women from the National Institutes of Health [16].

### Risk factor assessment

We considered multiple risk factors potentially relevant to periodontitis and osteoporosis based on previous work. The first category is the sociodemographic variables, including age (30–45, 45–60, > 60), gender, ethnicity, marital status, education level (high and below, college and above), poverty-income-ratio (PIR, categorized as low: < 1.00, normal and high: ≥ 1.00, lower PIR indicates higher poverty) and citizenship. The second category is lifestyle behaviors, such as smoking status (never, former and current), alcohol consumption (never, former, and current) and physical activity (low: ≤ 500 MET-min/week, normal: 500–1000 MET-min/week and high: ≥ 1000 MET-min/week). The third category is disease-related health conditions, including body Mass Index (BMI, undernutrition: < 18.5 kg/m^2^, normal weight: 18.5–24.9 kg/m^2^, overweight: 25–29.9 kg/m^2^, obese: ≥ 30 kg/m^2^), level of calcium (low: < 2.25 mmol/L, normal: 2.25–2.75 mmol/L, high: > 2.75 mmol/L), level of vitamin D (levels of 25-hydroxyvitamin D3 and 25-hydroxyvitamin D2, low: < 50 nmol/L, normal: 50–250 nmol/L, high: > 250 nmol/L), diabetes (no, prophase: impaired fasting glycaemia or impaired glucose tolerance, yes), hypertension (no, yes), Cardiovascular disease (CVD) such as coronary heart disease (no, yes), congestive heart failure (no, yes), heart attack (no, yes), angina (no, yes) and stroke (no, yes), cancer history (no, yes), arthritis (no, yes), hyperlipidemia (no, yes), patient health questionnaire-9 (phq-9: none, mild, moderate, moderately severe, severe), Parkinson (no, yes), viral hepatitis (no, yes), along with family history of osteoporosis (no, yes), and use of estrogen (no, yes) in the subgroup of menopausal women. Oral examination includes sum of teeth loss (0–5, 6–10, 11–15, 16–20, 21–25, > 25) and the floss usage (never, occasionally: 1–3 times/week, frequently: 4–7 times/week) is the last category.

### Statistical analysis

We employed SPSS software (version 24.0) and R-software (version 4.2.1) to perform the statistical analyses. Chi-square tests were performed to compare differences in periodontal status associated with selected confounders. Compared with the normal group, binary logistic regression analysis was performed to investigate the association between the presence of periodontal disease/ severe periodontitis and osteoporosis in the whole group and the subgroup of menopausal women. Specifically, model 1 was the crude model. In model 2, sociodemographic variables were included to test if there existed any residual confounding effect of sociodemographic variables in the association of periodontal disease/ severe periodontitis and osteoporosis. Model 3 was adjusted for model 2 adjustments plus lifestyle behaviors. In model 4, disease-related health conditions were further included. Fully adjusted model 5 was additional adjusted for oral examination. Subsequently, we further analyzed the interactions of statistically significant covariates with osteoporosis in the fully adjusted model 5. ORs and 95% confidence intervals (CI) were calculated and the statistical significance was set to *p* < 0.05.

## Results

From a total of 20,712 participants enrolled in NHANES 2009–2010 and 2013–2014, 11,006 individuals were excluded because of missing information of periodontal condition, 3970 individuals were subsequently excluded because no necessary osteoporosis data was available. Therefore, 5736 participants were finally included in the study, which represented 111.7 million noninstitutionalized residents of the United States. A total of 519 records met the criteria of menopausal women aged 45–60 years for the subgroup analysis, which represented 11.2 million noninstitutionalized residents.

The comparison of population characteristics by periodontal status is shown in Table 1. The periodontal status distribution of the whole participants was as follow: normal: 2746 (47.9%), mild: 326 (5.7%), moderate: 2020 (35.2%) and severe: 644 (11.2%). The distribution of BMD distribution was as follow: normal: 3620 (63.1%), osteopenia: 1863 (32.5%) and osteoporosis: 253 (4.4%). We found that the rates of normal BMD in the periodontal disease group and the severe periodontitis group were 60.65% and 65.53%, respectively. There were significant differences between normal group and periodontal disease group according to BMD, age, gender, ethnicity, marital status, level of education, level of income, citizenship, smoking status, alcohol consumption, BMI, level of calcium, level of vitamin D, diabetes, hypertension, coronary heart disease, congestive heart failure, heart attack, arthritis, Parkinson, viral hepatitis, family history, sum of teeth lost and floss usage. Except confounders of BMD, physical activity, BMI, level of calcium, arthritis and Parkinson, the distribution of severe periodontitis was similar to the group of periodontal disease (*p* < 0.05). And the reproductive health status of menopausal women can be seen in Supplemental Table.

**Table 1:** General characteristics and the presence of periodontal status (periodontal disease and severe periodontitis)

Variables	Normal n/N (%)	Periodontal disease n/N (%)	*P*-value	Severe periodontitis n/N(%)	*P*-value
**Bone mineral density**			**0.04**		0.54
normal	1755/40.7 m (63.32)	1864/28.8 m (60.65)		427/5.6 m (65.53)	
osteopenia	884/21.5 m (33.51)	978/16.5 m (34.65)		194/2.6 m (30.83)	
osteoporosis	106/2.0 m (3.17)	147/2.2 m (4.70)		22/312.8t (3.64)	
**Age**	50.76 ± 0.63	56.07 ± 0.80	** < 0.0001**	55.88 ± 0.90	** < 0.0001**
**Gender**			** < 0.0001**		** < 0.0001**
male	1131/28.2 m (43.86)	1790/28.3 m (59.54)		461/6.2 m (72.10)	
female	1614/36.1 m (56.14)	1199/19.2 m (40.46)		182/2.4 m (27.90)	
**Ethnicity**			** < 0.0001**		** < 0.0001**
white	1486/49.5 m (77.09)	1174/29.5 m (62.09)		203/4.6 m (53.39)	
black	379/4.6 m (7.22)	633/6.3 m (13.31)		168/1.6 m (18.80)	
mexican	330/3.2 m (5.02)	622/5.4 m (11.29)		163/1.2 m (13.63)	
other	550/6.9 m (10.68)	560/6.3 m (13.31)		109/1.2 m (14.17)	
**Marital status**			** < 0.0001**		** < 0.0001**
married	1769/45.4 m (70.73)	1692/28.1 m (59.27)		353/4.7 m (54.90)	
unmarried	975/18.8 m (29.27)	1294/19.3 m (40.73)		290/3.9 m (45.10)	
**Level of education**			** < 0.0001**		** < 0.0001**
middle and low	417/6.1 m (9.57)	990/11.2 m (23.54)		277/3.0 m (35.34)	
high and above	2325/58.0 m (90.43)	1992/36.3 m (76.46)		366/5.6 m (64.66)	
**Level of income**			** < 0.0001**		** < 0.0001**
below average	296/3.8 m (6.26)	630/7.0 m (15.81)		161/1.7 m (21.50)	
above average	2229/56.3 m (93.74)	2079/37.0 m (84.19)		410/6.1 m (78.50)	
**Citizenship**			** < 0.0001**		** < 0.0001**
no	291/3.8 m (5.94)	544/5.9 m (12.48)		130/1.3 m (15.41)	
yes	2451/60.3 m (94.06)	2435/41.5 m (87.52)		513/7.3 m (84.59)	
**Smoking status**			** < 0.0001**		** < 0.0001**
never	1761/41.5 m (64.57)	1405/21.8 m (45.78)		246/3.2 m (36.73)	
former	659/15.6 m (24.33)	859/14.6 m (30.73)		160/2.1 m (24.95)	
now	325/7.1 m (11.10)	723/11.2 m (23.49)		237/3.3 m (38.32)	
**Alcohol consumption**			** < 0.001**		**0.01**
never	324/5.7 m (9.24)	377/4.8 m (10.70)		68/732.5t (8.91)	
former	374/7.6 m (12.24)	588/8.0 m (17.71)		126/1.6 m (19.27)	
now	1929/48.5 m (78.52)	1860/32.4 m (71.59)		418/5.9 m (71.82)	
**Physical activity**			0.55		**0.004**
low	396/9.2 m (17.98)	390/6.1 m (16.85)		55/683.8t (10.58)	
normal	322/7.4 m (14.42)	298/5.0 m (13.76)		69/905.5t (14.01)	
high	1414/34.7 m (67.60)	1491/25.2 m (69.39)		339/4.9 m (75.42)	
**Body Mass Index**			**0.04**		0.06
undernutrition	20/512.3t (0.80)	37/600.3t (1.26)		9/174.9t (2.04)	
normal	743/18.4 m (28.77)	740/11.8 m (24.78)		154/2.1 m (24.54)	
overweight	993/23.2 m (36.16)	1115/17.8 m (37.50)		254/3.4 m (39.69)	
obese	985/22.0 m (34.27)	1088/17.3 m (36.45)		224/2.9 m (33.73)	
**Calcium**			**0.05**		0.3
normal	2481/56.9 m (94.26)	2650/42.5 m (92.78)		579/7.9 m (94.66)	
low	3163/.7 m (5.73)	225/3.2 m (7.03)		38/436.0t (5.22)	
High	1/6.9t (0.01)	6/85.4t (0.19)		1/10.2t (0.12)	
**Vitamin D**			** < 0.0001**		** < 0.0001**
normal	2078/52.9 m (83.88)	1959/33.7 m (72.95)		389/5.6 m (66.96)	
low	595/10.2 m (16.12)	943/12.5 m (26.99)		232/2.8 m (33.04)	
high	0/0 (0.00)	2/24.8t (0.05)		0/0 (0.00)	
**Diabetes**			** < 0.0001**		** < 0.001**
no	2141/52.4 m (81.61)	2024/34.5 m (72.46)		412/6.2 m (71.96)	
prophase	213/4.7 m (7.31)	272/4.0 m (8.40)		69/780.8t (9.08)	
DM	391/7.1 m (11.09)	693/9.1 m (19.13)		162/1.6 m (18.95)	
**Hypertension**			** < 0.0001**		** < 0.0001**
no	1683/40.9 m (63.62)	1461/24.8 m (52.06)		398/4.4 m (51.18)	
yes	1062/23.4 m (36.38)	1528/22.8 m (47.94)		345/4.2 m (48.82)	
**Coronary heart disease**			** < 0.001**		**0.05**
no	2679/62.9 m (97.98)	2848/45.4 m (95.83)		617/8.3 m (96.48)	
yes	63/1.3 m (2.02)	127/2.0 m (4.17)		26/303.0t (3.52)	
**Congestive heart failure**			** < 0.0001**		**0.01**
no	2721/63.8 m (99.33)	2907/46.5 m (97.94)		626/8.4 m (97.96)	
yes	22/431.4t (0.67)	73/975.8t (2.06)		15/175.2t (2.04)	
**Heart attack**			** < 0.0001**		** < 0.0001**
no	2699/63.3 m (98.54)	2838/45.4 m (95.49)		606/8.1 m (94.57)	
yes	44/939.4t (1.46)	146/2.1 m (4.51)		36/466.3t (5.43)	
**Angina**			0.15		0.77
no	2704/63.3 m (98.60)	2913/46.5 m (98.00)		625/8.4 m (98.45)	
yes	40/901.5t (1.40)	64/949.9t (2.00)		14/132.9t (1.55)	
**Stroke**			0.12		0.12
no	2678/62.7 m (97.76)	2876/46.1 m (97.06)		616/8.3 m (96.18)	
yes	64/1.4 m (2.24)	109/1.4 m (2.94)		26/327.7 m (3.82)	
**Cancer**			0.18		0.14
no	2477/57.1 m (88.88)	2645/41.3 m (87.01)		585/7.8 m (91.31)	
yes	268/7.1 m (11.12)	340/6.2 m (12.99)		58/746.9t (8.69)	
**Arthritis**			**0.002**		0.24
no	2010/47.3 m (73.65)	2054/32.7 m (68.87)		475/6.6 m (76.55)	
yes	730/16.9 m (26.35)	924/14.8 m (31.13)		166/2.0 m (23.45)	
**Hyperlipidemia**			0.11		0.83
no	730/17.0 m (26.43)	697/11.4 m (23.86)		168/2.2 m (25.97)	
yes	2015/47.2 m (73.57)	2292/36.2 m (76.14)		475/6.4 m (74.03)	
**Phq-9**			0.09		0.39
none	2029/49.3 m (79.81)	2152/35.0 m (77.34)		463/6.4 m (78.30)	
mild	382/8.6 m (13.90)	416/6.7 m (14.80)		96/1.1 m (14.02)	
moderate	119/2.1 m (3.43)	161/2.4 m (5.21)		29/436.9t (5.34)	
moderately severe	67/1.2 m (1.91)	62/896.2t (1.98)		12/107.5t (1.31)	
severe	23/586.4t (0.95)	28/307.2t (0.68)		10/84.0t (1.03)	
**Parkinson**			** < 0.001**		0.56
no	2733/64.0 m (99.60)	2946/46.8 m (98.36)		639/8.5 m (99.42)	
yes	12/258.7t (0.40)	107/778.7t (1.64)		4/50.2t (0.58)	
**Viral hepatitis**			** < 0.0001**		** < 0.001**
no	2624/62.3 m (98.77)	2794/44.4 m (96.26)		591/8.0 m (95.36)	
yes	46/775.0t (1.23)	107/778.7t (3.74)		30/389.2t (4.64)	
**Family history**			**0.03**		** < 0.001**
no	2255/51.5 m (83.81)	2554/39.0 m (86.86)		573/7.6 m (93.29)	
yes	373/10.0 m (16.19)	266/5.9 m (13.14)		38/549.5t (6.71)	
**Sum of teeth lost**	2.57 ± 0.21	5.49 ± 0.43	** < 0.0001**	6.68 ± 0.68	** < 0.0001**
**Floss usage**	3.72 ± 0.11	3.21 ± 0.18	** < 0.001**	2.59 ± 2.88	** < 0.0001**

Significant associations are bolded, N: weighted total population in United States, m: million, t: thousand

Table 2 and 3 showed the results of binary logistic regression analysis performed to investigate the association between periodontal disease/ severe periodontitis and osteoporosis in the whole group. In crude model 1, osteoporosis was significantly associated with an increased risk of periodontal disease (OR: 1.55, 95% CI: 1.17–2.04). After progressively adjusting for all included covariates in model 5, the adjusted OR was 1.66 (95% CI: 1.00–2.77) in participants with osteoporosis. Results showed that compared with the group of no periodontitis, periodontal disease was significantly associated with osteoporosis in participants who were unmarried black or Mexican male, with advanced age, abnormal level of vitamin D, history of smoking, Parkinson and multiple missing teeth, while citizenship, the habit of flossing, higher level of income and education were statistically significant in protecting against periodontal diseases, after analyzing the interactions of statistically significant covariates with osteoporosis in the fully adjusted model (*p* < 0.05). However, there existed no significant association between severe periodontitis and osteoporosis in the whole group.

**Table 2:** Association between periodontal disease and osteoporosis in the whole population

Variable	Model 1		Model 2				Model 3				Model 4				Model 5			
	**OR**	**95% CI**	**OR**		**95% CI**		**OR**		**95% CI**		**OR**	**95% CI**			**OR**		**95% CI**	
**Bone mineral density**																		
normal	ref	ref	ref		ref		ref		ref		ref	ref			ref		ref	
osteopenia	1.08	0.91–1.27	1.01		0.84–1.21		1.05		0.85–1.31		1.09	0.87–1.37			1.08		0.86–1.36	
osteoporosis	**1.55**	1.17–2.04**	1.24		0.85–1.80		1.40		0.84–2.32		**1.65**	1.02–2.66*			**1.66**		1.00–2.77*	
**Age**																		
< 45															ref		ref	
45–60															**2.02**		1.53–2.65***	
> 60															**3.47**		2.38–5.07***	
**Gender**																		
male															ref			
female															**0.51**		0.40–0.63***	
**Ethnicity**																		
white															ref			
black															**1.60**		1.10–2.31*	
mexican															**1.90**		1.40–2.59***	
other															1.39		0.98–1.96	
**Marital status**																		
married															ref			
unmarried															**1.32**		1.06–1.66**	
**Education**																		
middle and low															ref			
high and above															**0.54**		0.40–0.72***	
**Level of income**																		
low															ref		ref	
normal and high															**0.65**		0.47–0.88**	
**Citizenship**																		
no															ref			
yes															**0.68**		0.49–0.95*	
**Smoking status**																		
never															ref			
former															**1.65**		1.33–2.04***	
now															**2.83**		2.13–3.76***	
**Vitamin D**																		
normal															ref		ref	
low															**1.3**		1.04–1.62*	
high															5.1*10^**4**^		6.0*10^**3**^–4.3*10^5^***	
**Parkinson**																		
No															ref		ref	
yes															**5.06**		1.85–13.84**	
**Sum of teeth lost**																		
0–5															ref		ref	
5–10															**2.90**		2.10–3.99***	
10–15															**3.20**		1.82–5.64***	
15–20															1.66		0.91–3.05	
20–25															2.08		0.94–4.58	
> 25															1.55		0.50–4.80	
**Floss usage**																		
0																ref		
1–3																**0.67**		0.52–0.88**
4–7																**0.69**		0.52–0.90**

Model 1: crude. Model 2, adjusted for sociodemographic variables. Model 3: model 2 + lifestyle behaviors. Model 4: model 3 + disease-related health conditions. Model 5: model 4 + oral examination, fully adjusted model

Significant associations are bolded

*p < 0.05

^**^p < 0.01

^***^ *p* < 0.001

**Table 3:** Association between severe periodontitis and osteoporosis in the whole population

Variable	Model 1		Model 2		Model 3		Model 4		Model 5	
	**OR**	**95% CI**	**OR**	**95% CI**	**OR**	**95% CI**	**OR**	**95% CI**	**OR**	**95% CI**
**Bone mineral density**										
normal	ref	ref	ref	ref	ref	ref	ref	ref	ref	ref
osteopenia	0.89	0.69–1.15	0.80	0.56–1.15	0.78	0.52–1.16	0.81	0.55–1.19	0.79	0.53–1.12
osteoporosis	1.11	0.62–1.98	0.96	0.43–2.14	0.74	0.25–2.20	0.86	0.27–2.71	1.09	0.34–3.54

Model 1: crude. Model 2, adjusted for sociodemographic variables. Model 3: model 2 + lifestyle behaviors. Model 4: model 3 + disease-related health conditions

Model 5: model 4 + oral examination, fully adjusted model. Significant associations are bolded

*p < 0.05

^**^p < 0.01

^***^ *p* < 0.001

As shown in Table 4, there existed no significant association between periodontal disease and osteoporosis in the subgroup of menopausal women. Table 5 demonstrated that compared with the whole group, menopausal women with osteoporosis tended to have a lower risk of developing severe periodontitis in crude model 1, although such pattern was not statistically significant. However, the relationship was strengthened and turned significant after adjusting for all potential confounders (model 2–5). In the fully adjusted model 5, osteoporosis group had an adjusted OR of 9.66 (95% CI: 1.13–82.38) for developing severe periodontitis. After analyzing the interactions of statistically significant covariates with osteoporosis in the fully adjusted model 5, compared with the group of no periodontitis, severe periodontitis was significantly associated with osteoporosis in those who were smoker, with history of angina and multiple missing teeth, while citizenship was statistically significant in protecting against severe periodontitis (*p* < 0.05).

**Table 4:** Association between periodontal disease and osteoporosis in the subgroup of menopausal women

Variable	Model 1		Model 2		Model 3		Model 4		Model 5	
	**OR**	**95% CI**	**OR**	**95% CI**	**OR**	**95% CI**	**OR**	**95% CI**	**OR**	**95% CI**
**Bone mineral density**										
normal	ref	ref	ref	ref	ref	ref	ref	ref	ref	ref
osteopenia	0.76	0.48–1.21	0.78	0.48–1.25	1.04	0.53–2.02	1.42	0.67–2.99	1.47	0.66–3.28
osteoporosis	0.67	0.27–1.69	0.65	0.23–1.85	1.36	0.29–6.40	1.46	0.32–6.53	1.48	0.34–6.47

Model 1: crude. Model 2, adjusted for sociodemographic variables. Model 3: model 2 + lifestyle behaviors. Model 4: model 3 + disease-related health conditions

Model 5: model 4 + oral examination, fully adjusted model. Significant associations are bolded

*p < 0.05

^**^p < 0.01

^***^ *p* < 0.001

**Table 5:** Association between severe periodontitis and osteoporosis in the subgroup of menopausal women

Variable	Model 1		Model 2		Model 3		Model 4		Model 5	
	**OR**	**95% CI**	**OR**	**95% CI**	**OR**	**95% CI**	**OR**	**95% CI**	**OR**	**95% CI**
**Bone mineral density**										
normal	ref	ref	ref	ref	ref	ref	ref	ref	ref	ref
osteopenia	0.88	0.42–1.84	1.17	0.45–3.04	1.56	0.45–5.37	**6.00**	1.01–36.62*	**21.83**	1.30–367.60*
osteoporosis	0.71	0.07–7.39	0.88	0.12–6.42	1.53	0.12–20.12	4.33	0.32–58.36	**9.66**	1.13–82.38*
**Citizenship**										
no									ref	
yes									**0.01**	0.00–0.27**
**Smoking status**										
never									ref	ref
former									1.39	0.12–16.82
now									**263.11**	**8.83–7.8 × 10**^3^**
**Angina**										
no									ref	ref
yes									**6.7 × 10**^**16**^	**1.6 × 10**^**13**^**–2.8 × 10**^20^***
**Sum of teeth lost**									**1.2**	1.02–1.41*
0–5									ref	ref
5–10									3.74	0.25–54.90
10–15									**23.78**	1.06–536.07*
15–20									1.26	0.05–34.48
20–25									**81.05**	**2.55–2.6 × 10**^3^**
> 25									1.33	0.51–2.89

Model 1: crude. Model 2, adjusted for sociodemographic variables. Model 3: model 2 + lifestyle behaviors. Model 4: model 3 + disease-related health conditions

Model 5: model 4 + oral examination, fully adjusted model. Significant associations are bolded

*p < 0.05

^**^p < 0.01

^***^ *p* < 0.001

## Discussion

This nationally, representative sample-based NHANES survey that adjusted for varied confounders revealed that a possible association between periodontal disease and osteoporosis. Our results are consistent with some previous researches designed to elucidate the link between these two diseases. One possible link between them is that systemic bone resorption of osteoporosis may also closely correlated with the osteoporotic change of jaws and loss of alveolar bone. Also, decrease of mandibular cortical width and thinning of trabecular maxillary bone could act on the systemic change of BMD [17], which could constitute a “weakened resistance” of the periodontium to infectious challenge as to amplify the alveolar bone loss in periodontitis [18]. On the other hand, pro-inflammatory mediators such as IL-1, IL-6, RANKL and TNF-α have been found in both diseases, and they could play critical roles on the differentiation and activity of osteoclasts via NF-κB signaling, which may be the central mechanistic link of these two diseases [19]. Inhibition of the signaling could impair osteoclastic bone resorption and promote bone formation, which had been demonstrated in animal models of both diseases [20, 21]. Other than that, these mediators could act locally to impair the tissue response to periodontitis, as to aggravate the inflammatory response and accelerate systemic bone resorption by regulating host response in turn [22].

Generally, periodontitis affects more men [23] while osteoporosis affects more women [24]. Our results revealed that a larger proportion of males in the group of periodontal disease/ severe periodontitis (59.54%/ 72.10%) than the normal group (43.86%). While in the analysis of subgroup, menopausal women with osteoporosis were nearly 9 times to develop severe periodontitis than the whole population. It may be explained by the drop of estrogen production in the ovarian and the increase of testosterone, which are characteristics of menopause [6]. Estrogen is important to the balance of bone homeostasis for it could enhance bone formation by increasing osteogenic differentiation of mesenchymal stem cells, as well as induces osteoclast apoptosis by inhibiting osteoclast formation [25]. What’s more important, the role of estrogen in inflammation is receiving increasing attention. Estrogen deficiency associates with increased expression of inflammatory cytokines such as IL-1, IL-6 and TNFα, as well as decreased level of anti-inflammatory cytokines such as OPG and IL-10 [26, 27], which may also lead to damage of periodontal tissues [28].

Compared with the whole group, smoking status seemed to have greater impact on severe periodontitis in menopausal women with osteoporosis, which could also be explained by the features of menopause somewhat. According to the study of Gu et al., in the group of menopausal women, smokers had lower levels of total estrogen metabolites than non-smokers [29]. Given the important role of estrogen in periodontitis, it is understandable that smoking menopausal women with osteoporosis are more likely to develop periodontitis. Therefore, hormone replacement therapy (HRT) can be a novel approach to treat menopausal women with periodontitis, as it can improve mandibular bone density, reduce gingival bleeding and prevent teeth lost in humans [30, 31]. In addition, since menopause is often associated with dyslipidemia, increased blood pressure, increased sympathetic tone, endothelial dysfunction and vascular inflammation, it compounds many traditional CVD risk factors as well [32]. As our results showed that menopausal women with angina were at an amazing risk for severe periodontitis, which could be explained by reduced nitric oxide (NO) production due to changes of estrogen during such period, with dysfunction in endothelium-dependent relaxation, anti-inflammatory effects, antihypertrophic and antithrombotic activities. Although the role of NO in periodontitis is controversial, NO enables gingival epithelial cells to protect tissue from infection as to aid in the maintenance of homeostasis in gingival circulation [33]. What’s more, elevated levels of NO could inhibit bone resorption through the apoptosis of bone marrow osteoclast progenitor cells and inhibition of mature osteoclast reabsorption activity [34]. Similarly, HRT could enhance local NO production and rectify NO deficiency in postmenopausal women [35]. Nevertheless, our results showed that the risk of severe periodontitis was reduced with the use of estrogen, though they’re not statistically significant. This may be due to the limitation of the sample size and survey design, we cannot track more details such as the level of estrogen, recommended dosage and daily usage of it. Moreover, estrogen may play roles of both pro-inflammatory and anti-inflammatory depending on different physiologic context [35, 36].

Meanwhile, our results highlighted the influence of socioeconomic factors on the development of periodontal disease. The probability of periodontitis in participants was expected to increase as the population advanced in age. Among the elderly, in addition to frequent accumulation of pathogens and reduced self-cleaning of saliva, accumulation of oxidative stress and cellular senescence are more important reasons related to the impairment of periodontal homeostasis [20, 42]. However, the OR of developing periodontal disease was lower in those who were married citizen with higher levels of education and income, for they were generally supposed to have easier access of oral examination. Vitamin D deficiency is another risk factor of periodontal disease since such microelement could inhibit secretion of inflammatory cytokines and downregulate adaptive immunity (T and B cells) [37, 38]. Whereas, subjects with high level of vitamin D were also susceptible to periodontitis, for it did nothing to improve bone density but increase the risk of fracture with the suppression effect on PTH, which could active osteoclasts and reduce bone formation [39, 40].

It's worth noting that we introduced the risk factor of the sum of teeth lost, which is important for the assessment of the periodontal status and has not been mentioned in previous cross-section study [28, 41]. Compared with other clinical measures, it’s may more sensitive since PD and CAL appear to depend mainly on the teeth retained, while most osteoporotic patients present with premature tooth loss [5, 42]. Moreover, tooth loss may be a cumulative result of progressing periodontal pockets and attachment loss. This may be one reason why some researches failed to find a positive association between osteoporosis and periodontitis when they used other indicators for adjustment [8, 43].

Nonetheless, prevention is more reasonable than treatment. Dentists are more likely to achieve success in treating periodontitis when they help patients with early diagnosis, prevention and treatment of osteoporosis, which is also consistent with the concept of personalized periodontology. Similarly, another cross-sectional study found that postmenopausal women receiving osteoporosis treatment had less periodontal probing depth, clinical attachment and gingival bleeding than participants that didn’t receive any treatment [44]. Besides, habit of flossing, routine oral examination and BMD screening are recommended for menopausal women, and regular dental images could also serve as a low-cost screening tool for early diagnosis of osteoporosis [5]. Moreover, healthy lifestyles such as quitting smoking, prevention of systemic diseases such as Parkinson and CVDs, and necessary supplement of vitamin D and estrogen are recommended, especially for menopausal women.

### Limitations

1. As it’s a cross-sectional study, selection bias was inevitable. Also, the temporality between periodontitis and osteoporosis was unclear, thus we cannot determine the causality.
2. There are still some problems such as inaccurate periodontal examination, incomplete information about periodontal examination and questionnaires. More detailed information and rigorous design should be provided ideally.
3. Some extreme values existed in our results, especially in the subgroup analysis. It may imply the association between periodontitis and osteoporosis in menopausal women could be different from that in the general population, and clinicians should be careful about the close relationship between these two diseases in menopausal women. Such result might be caused by the insufficient sample number as well as uncertainties in various aspects of menopause, and should be interpreted with caution. More high-quality studies with large samples are expected in the future.

## Conclusions

The significance of our research lies in the fact that: 1) For the first time, we used a large representative sample of NHANES to assess the association between periodontitis and osteoporosis, and our results showed that osteoporosis is significantly associated with periodontitis and the association is even more pronounced in menopausal women with severe periodontitis. 2) Compared with other cross-sectional studies, we have adjusted for a more comprehensive range of confounders. In the future, more rigorous, large sample and well-controlled longitudinal studies are expected.

## Supplementary Information

**Additional file 1: Supplemental Table** Reproductive health status of menopausal women.

## Data Availability

The datasets generated and analysed during the current study are available in the website of the National Health and Nutrition Examination Survey (NHANES) at https://www.cdc.gov/nchs/nhanes/index.htm.

## Abbreviations

NHANES: The National Health and Nutrition Examination Survey
BMI: Body mass index
BMD: Bone mineral density
OR: Odds ratio
PD: Pocket depth
AL: Attachment loss
CDC/AAP: The Centers for Disease Control and Prevention and American Academy of Periodontology
DXA: Dual-energy radiation absorptiometry
CI: Confidence intervals
CVD: Cardiovascular disease
phq-9: Patient health questionnaire-9
HRT: Hormone replacement therapy
NO: Nitric oxide
