# Supplementary material for: Association between periodontitis and osteoporosis in United States adults from the National Health and Nutrition Examination Survey: a cross-sectional analysis
Source: BMC Oral Health. 2023 May 2;23:254. doi: 10.1186/s12903-023-02990-4 (PMC10155350; doi:10.1186/s12903-023-02990-4)
Supplement: Supplementary file 1 — Additional file 1: Supplemental Table Reproductive health status of menopausal women. [file 12903_2023_2990_MOESM1_ESM.docx]

| Covariates of reproductive health | Mean ± SD/ Percentage | |
| --- | --- | --- |
| Age at menarche | 12.88 ± 1.77 | |
| Age at menopause | 46.49 ± 6.53 | |
| Number of pregnancies | 3.28 ± 1.81 | |
| Age at first childbirth | 22.71 ± 5.28 | |
| Age at last childbirth | 30.14 ± 6.15 | |
| Hysterectomy | No: 400 (77.1%) | Yes: 117 (22.5%) |
| Oophorectomy | No: 461 (88.8%) | Yes: 56 (10.8%) |
| Use of hormone | No: 399 (77.2%) | Yes: 117 (22.5%) |

Supplemental Table. Reproductive health status of menopausal women
